# Spatial Distribution of Aboveground Carbon Stock of the Arboreal Vegetation in Brazilian Biomes of Savanna, Atlantic Forest and Semi-Arid Woodland

**DOI:** 10.1371/journal.pone.0128781

**Published:** 2015-06-12

**Authors:** Henrique Ferraco Scolforo, Jose Roberto Soares Scolforo, Carlos Rogerio Mello, Jose Marcio Mello, Antonio Carlos Ferraz Filho

**Affiliations:** 1 Department of Forestry and Environmental Resources, North Carolina State University, Raleigh, North Carolina, United States of America; 2 Department of Forest Science, Federal University of Lavras, Lavras, Minas Gerais, Brazil; 3 Department of Engineering, Federal University of Lavras, Lavras, Minas Gerais, Brazil; University of California Davis, UNITED STATES

## Abstract

The objective of this study was to map the spatial distribution of aboveground carbon stock (using Regression-kriging) of arboreal plants in the Atlantic Forest, Semi-arid woodland, and Savanna Biomes in Minas Gerais State, southeastern Brazil. The database used in this study was obtained from 163 forest fragments, totaling 4,146 plots of 1,000 m^2^ distributed in these Biomes. A geographical model for carbon stock estimation was parameterized as a function of Biome, latitude and altitude. This model was applied over the samples and the residuals generated were mapped based on geostatistical procedures, selecting the exponential semivariogram theoretical model for conducting ordinary Kriging. The aboveground carbon stock was found to have a greater concentration in the north of the State, where the largest contingent of native vegetation is located, mainly the Savanna Biome, with Wooded Savanna and Shrub Savanna phytophysiognomes. The largest weighted averages of carbon stock per hectare were found in the south-center region (48.6 Mg/ha) and in the southern part of the eastern region (48.4 Mg/ha) of Minas Gerais State, due to the greatest predominance of Atlantic Forest Biome forest fragments. The smallest weighted averages per hectare were found in the central (21.2 Mg/ha), northern (20.4 Mg/ha), and northwestern (20.7 Mg/ha) regions of Minas Gerais State, where Savanna Biome fragments are predominant, in the phytophysiognomes Wooded Savanna and Shrub Savanna.

## Introduction

The top carbon dioxide emitters in the world are China, USA, and India, with Brazil occupying the fourteenth place on the list [1]. In 2009, due to the increase in carbon emissions, the Brazilian government launched the National Policy on Climate Change [2]. Among other initiatives, this law proposed reducing greenhouse gases emissions up to 38.9%, compared to expected emissions for 2020 (total expected emissions for 2020 is 3.236 GtCO_2_eq). More than half of the total emissions reduction is expected to come from reducing deforestation in all Brazilian Biomes, showing that the identification and mapping of carbon stock is very important.

Brazil has a total area of 8,514,877 km², of which 7% occurs in Minas Gerais State, i.e., 586,528 km^2^, an area larger than France and Belgium together. The large area encompasses landscape variations ranging from Savanna, Atlantic Forest, and Semi-arid woodland, representing 57%, 41%, and 2% of the native vegetation, respectively. Authors [3] have reported the occurrence of at least 2,401 tree species containing diameter at breast height (DBH) > = 5 cm for these three distinct Biomes in Minas Gerais State.

In Minas Gerais State, the Savanna Biome provides landscape variations comprising phytophysiognomes from Dry Grassland to Densely Wooded Savanna. In Brazil, Savanna is the second largest Biome, second only to the Amazonian Biome. This Biome is also a world biodiversity hotspot, including many endemic and rare species [4]. The Atlantic Forest Biome found in Minas Gerais State is composed of phytophysiognomes Semideciduous Seasonal Forest and Rain Forest, the first with larger area. Atlantic Forest [5] is a Biome of great wealth of plant species, plant density, and endemic animals. These characteristics [4] placed this Biome among the eight most important hotspots for biodiversity conservation. In addition, in Minas Gerais State, Semi-arid woodland represents the phytophysiognomy of Deciduous Seasonal Forest. In Brazil, Semi-arid woodland is an ecosystem occupied by tropical dry forest and shrub vegetation. It also includes areas with sedimentary soils, called Semi-arid woodland sand [6], which occur only in Brazil and is almost fully restricted (98.8%) to the northeastern region of the country. Semi-arid woodland [7] regions are unique for being in areas with high temperature and low average rainfall, being characterized as the region with the greatest meteorological limitations in Brazil, due to its climatic variability.

To define public policies and strategies for the application of control instruments and spatial management of forest remnants, precise and high quality information is of utmost importance. The “Forest Inventory of Minas Gerais”, conducted by *Universidade Federal de Lavras* (Federal University of Lavras, UFLA), generated abundant information regarding the quality of forest remnants (fragments) in the State. It also includes information related to the determination of carbon stock and the ongoing monitoring of forests’ development throughout permanent plots. This important instrument enables the identification of the main factors and causes that lead to changes in land use in the State, which leads to the impoverishment of its biological diversity. Studies have been developed from this database, such as [8–9]. However, these studies were conducted in specific locations, and information is still lacking in regard to the knowledge of distribution of carbon stock in the various phytophysiognomes.

In this context, geostatistics with global prediction methods emerges as a highly suitable technique, as long as the sampling quantity and quality of the variable of interest is appropriate, because the quality of the parameterized spatial model is a direct function of the sampling [10]. The geostatistics field has a wide range of techniques that enable accurate estimates of the studied environmental variables. Apart from ordinary Kriging, Co-kriging, which is treated as a multivariate extension of Kriging [11], and Regression-kriging, which comprises a combination of geographic multivariate models and the stochastic behavior of Kriging [12], are alternatives for providing spatialization.

In Brazil, rainfall erosivity, soil attributes and environmental indicators associated with groundwater recharge [13–14] are examples of variables that have used mapping techniques that use the spatial approach combined with regression (Regression-kriging). However, for mapping carbon stock, particularly in vegetation, there is a lack of studies that can be used for public policies to meet society demands and contribute to a reduction in deforestation and land-use change.

The use of statistical models [15–16] for mapping quantities may provide good performance, because such models seek the relationship of cause and effect between the main variable and geographic variables, such as latitude, longitude, and altitude. However, [13] drew attention to the fact that the use of multivariate statistical models can produce either overestimated or underestimated results for certain sites whose input variables are close to the established limits, which can be corrected with the application of ordinary Kriging of residuals. Thus, it is plausible that Regression-kriging can produce better results among the interpolators best known for combining features of a multivariate statistical model, i.e., the relationship of cause and effect with geographic variables, with geostatistics (stochastic approach), which is applied for producing a prior map of residuals generated by the multivariate model. This means that the map estimated by the multivariate statistical model will be corrected based on the residual’s map, thus reducing distortions that may be produced by the map generated by a purely statistical model [14–15].

For the Brazilian scenario, there is a crucial scientific gap in understanding aboveground carbon stock distribution for large areas. This fact is even more relevant when considering the high deforestation rates in Brazil and in Minas Gerais State, putting at stake not only the existing biodiversity but also the capture and increase of carbon stock. In this context, this study evaluates aboveground carbon stock mapping of arboreal plants of this important Brazilian State, which is a geographic reference of the great vegetation diversity existing in Brazil.

Thus, the objective of this study was to map the spatial distribution of aboveground carbon stocks of arboreal plants (stem and branch wood for trees with DBH larger than 5cm) using Regression-kriging procedure, and subsequently describing carbon concentration distribution in aboveground parts of arboreal plants in the Atlantic Forest, Savanna, and Semi-arid woodland Biomes in Minas Gerais State.

## Materials and Methods

### Physiographic and social-economic characterization of Minas Gerais State

Minas Gerais is the fourth largest in area and the second most populous State in Brazil, with a population of 20.6 million, a demographic density of 33.4 people per square kilometer. In economic terms, Minas Gerais is the Brazilian State with the third largest gross domestic product (9.3% of the Brazilian GDP), corresponding a total of 175.7 billion dollars in 2010 [17].

**Fig 1:**
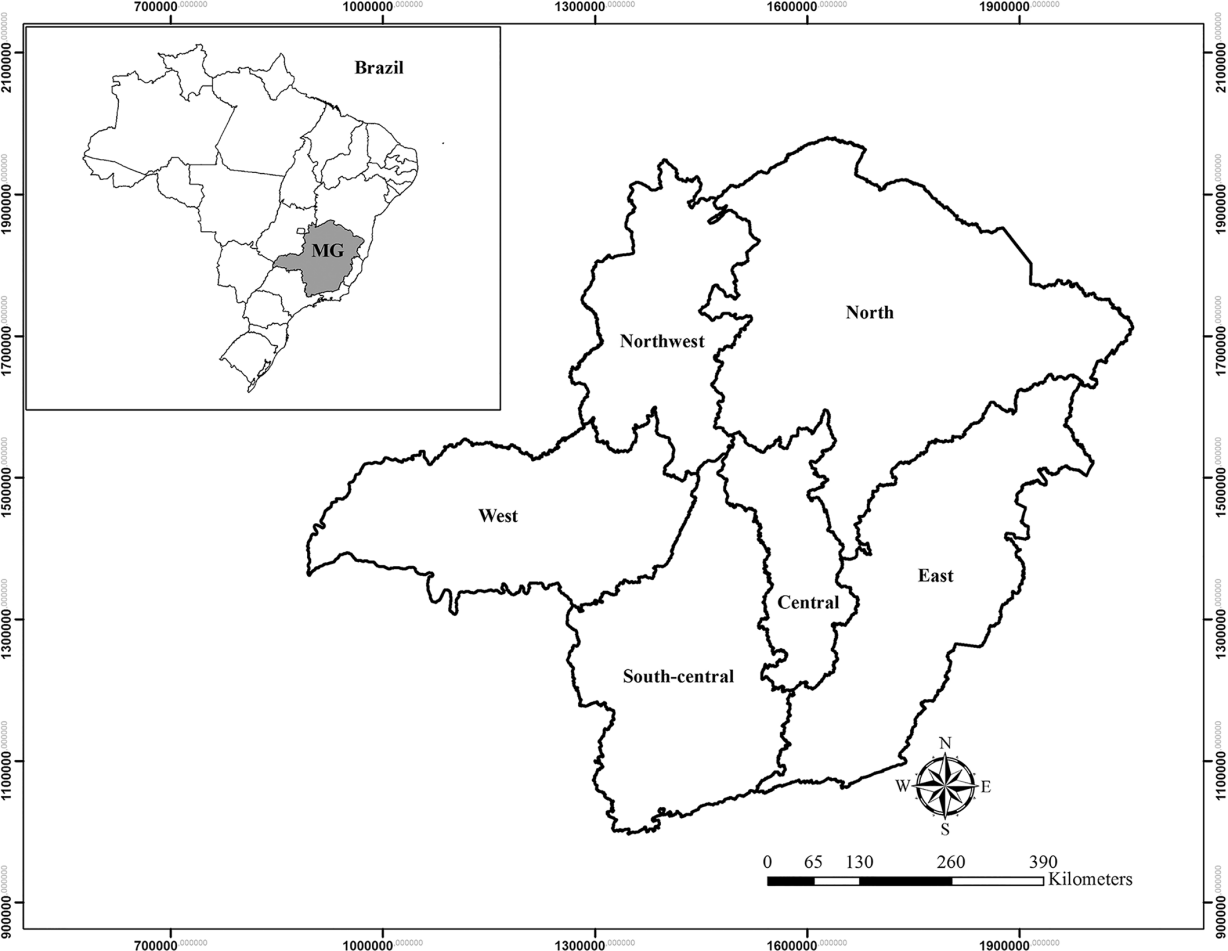
Location of Minas Gerais State in Brazil and its division into six regions.

Overall, Minas Gerais State may be divided into six macro-regions: central, south-central, east, northwest, north, and west (Fig 1).

**Fig 2:**
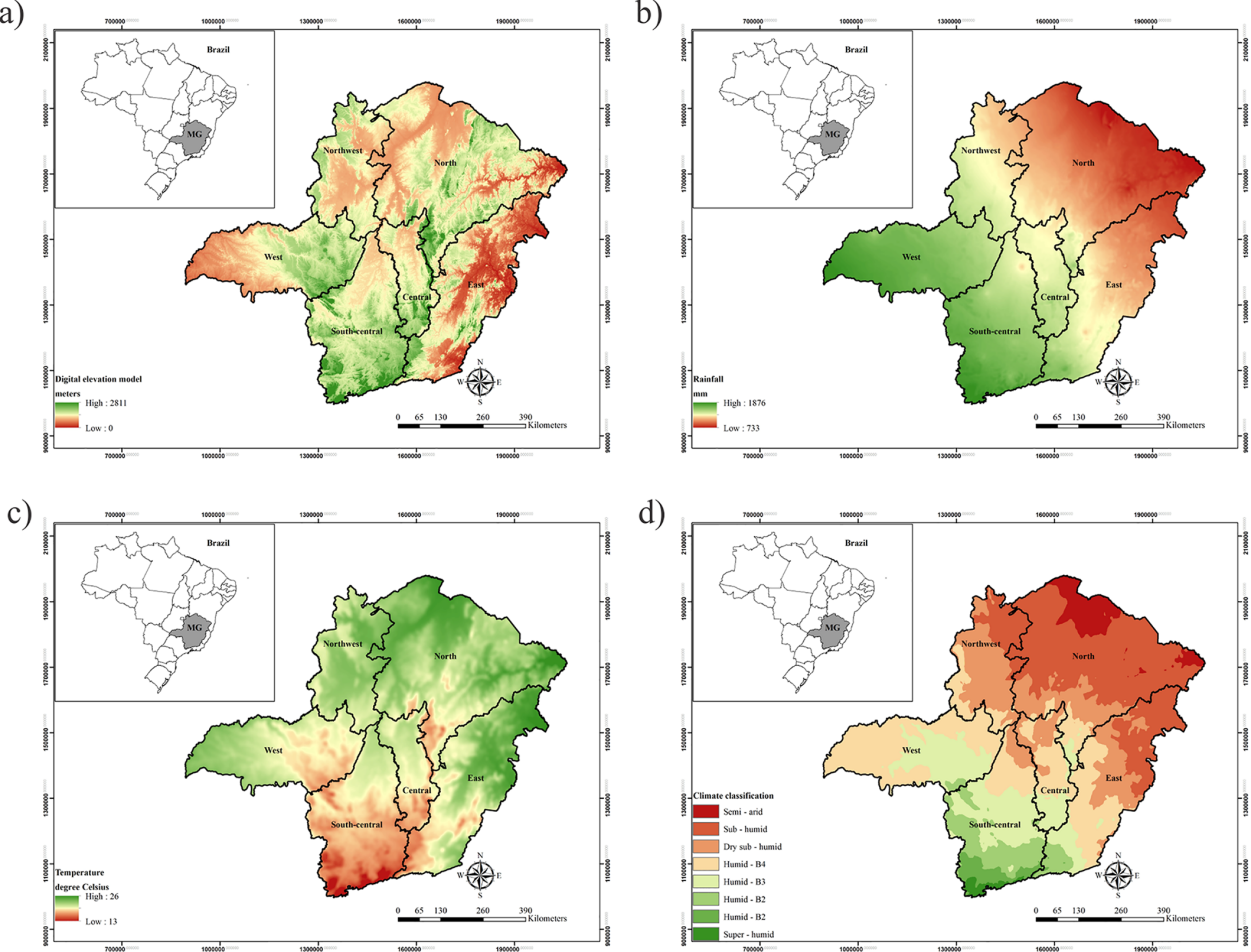
Topographic spatial distribution (a), average annual rainfall (b), average annual air temperature (c), and Thorntwaite climate classification (d) for Minas Gerais State.

Fig 2 presents the topographical characteristics of Minas Gerais State, exhibiting altitudes mostly varying between 900 to 1500 meters, except for the eastern region as there is a marked variability from the lowest altitudes of around 100 meters to the *Pico da Bandeira* with 2,891 meters (this is the highest point in the State and second highest of Brazil). The digital elevation model (DEM), which shows the topographical conditions of Minas Gerais State, was derived from the "Shuttle Radar Topography Mission" (NASA-SRTM), and a map matrix with a resolution of 100 m was developed. From this map, it is possible to extract the geographical variables latitude, longitude and altitude of each cell (pixel) on the territory of Minas Gerais State.

Furthermore, in Fig 2, it is possible to observe the base maps with the most important average climatic characteristics of the State as well as the climatic classification of the regions based on Thornthwaite methodology [18]. An effective climate variability in the State can be noticed, with negative rainfall and positive temperature gradients from south to north. Generally, altitude and latitude are the geographical elements with the greatest impact on climate [13]. This variability helps to explain the different Biomes and the phytogeographic characteristics of the State. Savanna is predominant in the west/northwest, with warmer and wetter climate during summer, and a pronounced dry period. Semi-arid woodland is predominant in the northern area, characterized by semi-arid and sub-humid climates, with higher temperatures and lower rainfall throughout the year. Finally, Atlantic Forest dominates the south and east regions, featuring more rainy and mild climates, particularly in the south.

### Sampling and stock determination of aboveground carbon of the arboreal individuals of the Biomes in Minas Gerais State

The database used in this study was obtained from 163 forest fragments of a multistage sampling methodology used in each hydrographic basin in the Semi-arid woodland, Savanna, and Atlantic Forest Biomes. The *Instituto Estadual de Florestas* (State Institute of Forests-IEF), which is the environmental institute of Minas Gerais State, is responsible for these fragments, since the areas are on public lands. Thus, the field data collection was approved by the responsible institute in Minas Gerais State.

**Fig 3:**
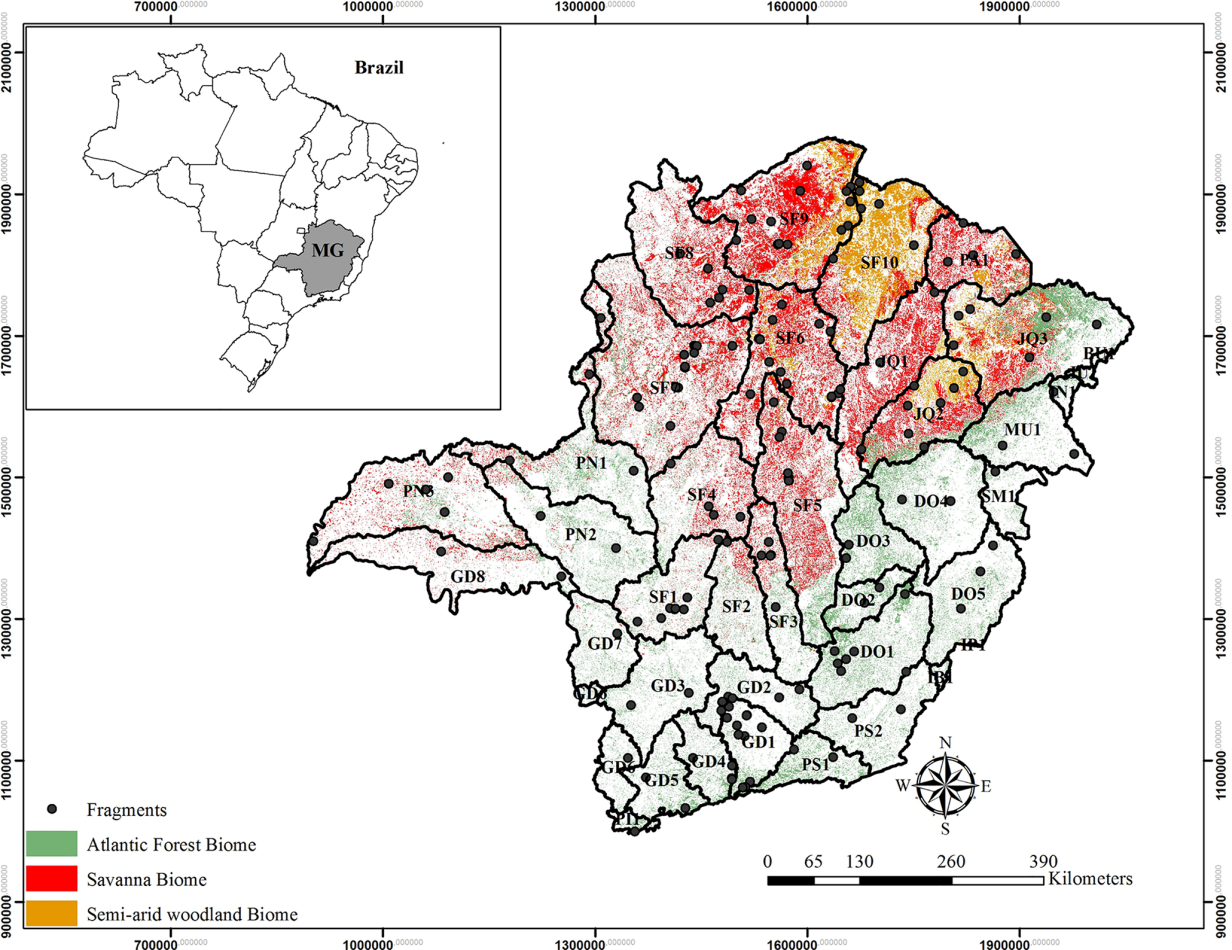
Geographic location of Minas Gerais State with spatial distribution of Biomes, subwatersheds and analyzed forest fragments.

The first step of the multistage sampling technique was to determine the areas of the State’s subwatersheds. These were not considered a sampling stage since all 40 subwatersheds were sampled. We then quantified the area of each forest type within these subwatersheds. The 4,146 sample plots were distributed proportionally to each subwatershed, being the proportion used the amount of vegetation area in the subwatershed in relation to the State’s total vegetation area (15,254,539 ha). The sample plots were distributed within each subwatershed proportionally to the area of each forest Biome with these subwatersheds. Thus, phytophysiognomy was defined as the first sampling stage. The second sampling stage were the random forest fragments within each phytophysiognomy and the third sampling stage the sample plots within each forest fragment. Each sampled forest fragment received plots of 10x100 meters. Each sample plot was distanced 1,000 by 600 meters from each other. The number of sample plots within each fragment was varied, since the sample plots were always distributed in the same distance from each other and the forest fragment size varied. Thus, the multistage sampling design had different sized first and second other units. Fig 3 shows the sampled forest fragments within each subwatershed of Minas Gerais State.

Thus, the database was obtained between 2006 and 2008, and the 4,146 permanent sample plots contain 780,000 trees with DBH > = 5cm. The sampling distribution for each Biome and phytophysiognomy was as follows: Savanna Biome, 61 forest fragments with 1,453 plots divided by: Shrub Savanna, 5 fragments with 188 plots; Wooded Savanna, 51 fragments with 1,108 plots; and Densely Wooded Savanna, 5 fragments with 157 plots. In the Semi-arid woodland Biome, 15 forest fragments were sampled with 571 plots in Deciduous Seasonal Forest. In the Atlantic Forest Biome, 79 fragments with 1,952 plots for Semideciduous Seasonal Forest and 8 fragments with 170 plots for Rain Forest, totaling 87 forest fragments sampled with 2,122 plots. The sampling intensity was sufficient for all sampled fragments, this was verified by applying linear-plateau segmented regression models [19].

The data used in the analysis was the average of the carbon stock of the plots contained for each of the 163 fragments, due to the heterogeneous nature of the forest fragments [20].

A high number of forest fragments were evaluated for each Biome. The fragments were selected trying to ensure that the whole spectrum of vegetation variation was sampled. Thus, there are forest fragments in different degrees of anthropization, different sites, different successional stages, and trees with different diameters and heights, leading to an increase in the variability of the vegetation [21–23]. In Table 1, average descriptive statistics for each Biome are shown, highlighting structural variability among them.

**Table 1:** Descriptive average statistics of the Biomes for Minas Gerais State.

Biome	Phytophysiognomy	V (m^3^/ha)	DW (Mg/ha)	N/ha	N° species
Savanna	Shrub Savanna	18.6	11.4	401	202
Savanna	Wooded Savanna	50.0	30.2	1,173	686
Savanna	Densely Wooded Savanna	117.5	56.3	1,649	317
Savanna	Average and No. of species	62.0	32.6	1,074	1,205
Atlantic Forest	Semideciduous Seasonal Forest	198.3	102.9	1,377	1336
Atlantic Forest	Rain Forest	279.4	135.3	1,864	513
Atlantic Forest	Average and No. of species	238.8	119.1	1,621	1,849
Semi-arid woodland	Deciduous Seasonal Forest	151.2	88.7	973	365

V is the volume per ha; DW is the dry matter weight per ha; N/ha is the number of individuals per hectare.

The trees used to determine carbon concentration were all from destructive sampling campains. For the three Biomes, 2,060 trees were scaled and divided into categories according to diameter and height, proportioned by the relative density of species sampled from the 163 forest fragments.

The diameter classes were composed of 5 cm classes starting from 5 cm, the height classes were composed of 2 m classes starting from 4 m height. The sampling consisted of 4 species that presented the highest relative density values within each of the 6 phytophysiognomes and the rest of the species within each subwatershed were grouped into a single group. The 4 species for each phytophysiognomy was as follows: Shrub Savanna, *Qualea grandiflora* Mart., *Eugenia dysenterica* DC., *Qualea parviflora* Mart., *Dalbergia miscolobium* Benth; Wooded Savanna, *Qualea parviflora* Mart., *Qualea grandiflora* Mart., *Eugenia dysenterica* DC., *Eriotheca pubescens* (Mart. & Zucc.) Schott & Endl; Densely Wooded Savanna, *Xylopia aromatica* (Lam.) Mart., *Qualea grandiflora* Mart., *Qualea multiflora* Mart., *Talisia esculenta* (A.St.-Hil.) Radlk.; Semideciduous Seasonal Forest, *Eremanthus incanus* (Less.) Less., *Copaifera langsdorffii* Desf., *Myrcia splendens* (Sw.) DC., *Tapirira obtusa* (Benth.) J.D.Mitch.; Rain Forest, *Psychotria vellosiana* Benth., *Myrsine umbellata* Mart., *Myrceugenia alpigena* (DC.) Landrum, *Myrcia splendens* (Sw.) DC.; Deciduous Seasonal Forest, *Handroanthus chrysotrichus* (Mart. ex A.DC.) Mattos, *Anadenanthera colubrina* (Vell.) Brenan, *Myracrodruon urundeuva* Allemão, *Machaerium acutifolium* Vogel. These were the control groups used to select the different trees for destructive sampling to determine biomass.

Scaling conducted in a total of 1,091 trees (distributed in 304 species) belonged to the Savanna Biome were divided into 915 from Wooded Savanna and Shrub Savanna, and 176 from Densely Wooded Savanna. For the Atlantic Forest Biome, scaling was conducted in 832 trees (distributed in 456 species), divided into 674 from Semideciduous Seasonal Forest and 158 from the Rain Forest. For the Semi-arid woodland Biome, 137 trees (distributed in 43 species) came from Semideciduous Seasonal Forest.

The destructive sampling was conducted by first measuring the DBH, total height and stem height prior to felling the tree. The tree was then georeferenced, cut down and had its height measured with a measuring tape. The tree was then scaled using Huber’s method.

Wood discs were sampled in each tree to determine dry weight and carbon content. These discs were taken at 0%, 25%, 50%, 75% and 100% of the height. Carbon percentage of each tree was determined based on the dry matter of different sections of each plant. The methods used for analyzing carbon were complete combustion, followed by reduction, chromatographic separation, and detection of products in a thermal conductivity detector [24]. From these data, equations for carbon (Eqs 1–5) were developed, which later were applied to the trees of each plot of the 163 sampled fragments. The equations were parameterized by phytophysiognomy, and enabled the calculation of carbon stock per fragment. It was tested the possibility of grouping all the data to fit one general equation to estimate tree carbon (using Graybill’s identity test), permitting the grouping only of Shrub Savanna and Wooded Savanna. The tests results determined the separation of the data to fit 5 different equations [8, 25] (Eqs 1–5):

Semi-arid woodland Biome

$$\begin{array}{l} C\text{ = }e^{\text{-10.7501678493+2.0580637328ln(}DBH\text{)+0.8604515609ln(}H\text{) }}\\ R^{2}\;=\;93.91\%\;and\;Syx\;=\;0.0013Mg \end{array}$$ (1)

Savanna Biome

Shrub Savanna and Wooded Savanna:

$$\begin{array}{l} C\text{ = }e^{\text{-11.1279639766+2.3816314802ln(}DBH\text{)+0.6106838246ln(}H\text{)}}\\ R^{2}=\;97.08\%\;and\;Syx\;=\;0.0025Mg \end{array}$$ (2)

Densely Wooded Savanna:

$$\begin{array}{l} C\text{ = }e^{\text{-10.8771683824+2.6359736325ln(}DBH\text{)+0.60878059946ln(}H\text{)}}\\ R^{2}=\;94.86\%\;and\;Syx\;=\;0.0057Mg \end{array}$$ (3)

Atlantic Forest Biome

Semiciduous Seasonal Forest:

$$\begin{array}{l} C\text{ = }e^{\text{-10.9520199234+2.0898526615ln(}DBH\text{)+0.809616224ln(}H\text{)}}\;\\ R^{2}=\;92.67\%\;and\;Syx\;=\;0.0100Mg \end{array}$$ (4)

Rain Forest:

$$\begin{array}{l} C\text{ = }e^{\text{-11.319842099+2.1415723631ln(}DBH\text{)+0.8134282561ln(}H\text{)}}\\ R^{2}=\;97.79\%\;and\;Syx\;=\;0.0027Mg \end{array}$$ (5)

Where: *C* is the stock of aboveground carbon (Mg) for each arboreal individual; e is the base of the natural logarithm; ln is the natural logarithm; *DBH* is the diameter measured at 1.30 meters above the ground (cm); and *H* is the total tree height (m); R^2^ is the coefficient of determination; Syx is the residual standard error.

### Mapping aboveground carbon stock of arboreal vegetation of Biomes in Minas Gerais State

Regression-kriging was applied for mapping aboveground carbon stock of the arboreal vegetation of Minas Gerais State. The methodology included the combination of two spatial interpolators, one global and the other stochastic. The first interpolator concerns the application of a geographic multiple linear regression model, which captures in a global way the behavior of the main variable, i.e., the one to be mapped. This interpolator produces a global map, enabling the identification of the general spatial behavior of the variable, without detailing more specific areas or regions [13]. For the final map to contain more details of specific areas, it is necessary to make a correction of the first map, developed exclusively based on a geographical model. Thus, ordinary Kriging was applied to the residuals generated by the geographic model to correct trends and detail the spatial behavior of the main variable, introducing the stochastic aspect to the mapping [13, 15]. It is expected that aboveground carbon stock of the arboreal vegetation is influenced by latitude and altitude since such geographic variables are correlated to climatic conditions in Minas Gerais State, as noted by [26]. This condition affects the Biomes of the State, and therefore can be geographically modeled.

For the parameterization of the geographic model, latitude (LA) and altitude (AL) data, extracted from the digital elevation model (DEM), and Biome as a categorical variable were used. To this end, the base model tested was as follows (Eq 6):

$$C=Biome+b_{1}\times LA+b_{2}\times \frac{1}{AL}$$ (6)

Where: *C* is carbon stock (Mg/ha); *Biome* is a categorical variable with 3 factors, i.e., Atlantic Forest, Savanna and Semi-arid woodland; *LA* is latitude in decimal degrees (Albers coordinates); *AL* is altitude in meters; and *b*_*i*_ are the parameters to be estimated by regression.

Eq 6 was fitted using R 3.0.0 software. This model was validated using auto-validation, where the precision statistic Mean Absolute Error (MAE) (Eq 7) was characterized, besides the residual standard error (Sxy) and the scatter plot of residuals. In addition, the Variance Inflation Factors (VIF) and Shapiro Wilk tests to check if the model presented multicollinearity and dependent residuals, respectively, were applied.

$$MAE(\%)=\frac{1}{n}\sum_{i=1}^{n}\left|\frac{O_{i}-P_{i}}{O_{i}}\right|\times 100$$ (7)

Where *O*_*i*_ and *P*_*i*_ are observed and predicted carbon stock values (Mg/ha), respectively; and *n* corresponds to the number of forest fragments.

From this process, the behavioral analysis of the spatial continuity of the residues derived from geographical model was done, i.e., studying their spatial autocorrelation as a function of distance. Theoretical semivariogram model was parameterized to the experimental semivariogram using R software [27], with the use of geoR package [28]. Thus, the map of ordinary Kriging of residuals was developed.

In this study, the exponential model has been applied, as described by [29], for obtaining the set of parameters to be used in the ordinary Kriging estimation of residuals. This model was chosen because in previous studies it has been shown to have a desirable performance for quantities such as rainfall mapping [30] and mapping of the aboveground carbon stock of Mexican conifer afforestation [31]. The exponential model parameterization was conducted by the method of weighted least squares.

For application of Regression-kriging, georeferenced continuous cells with dimensions of 100 × 100 m (1ha) were created throughout the length of the vegetation in Minas Gerais State using ArcMap [32]. In each of these cells, the latitude and altitude were characterized, and the geographic model was applied to them, generating a global map of carbon stock. The residual Kriging map was then added to the global map generated by the geographic model, thus generating the aboveground carbon stock map from the distribution of the arboreal vegetation of the Atlantic Forest, Savanna, and Semi-arid woodland Biomes in Minas Gerais State.

## Results and Discussion

### Aboveground carbon stock modeling in the arboreal vegetation in Biomes of Minas Gerais State

Pearson’s correlation coefficients between carbon stock and latitude, longitude, and altitude variables are presented in Table 2. This is an important step for setting a consistent geographical modeling, since this test directs which geographic variables may have a significant impact on the relationship with the dependent variable.

**Table 2:** Pearson’s correlation coefficients between aboveground carbon stock of the arboreal vegetation in Minas Gerais State (Mg/ha) and the variables latitude, longitude, and altitude.

Variable	Latitude	Longitude	Altitude
Carbon Stock	-0.5187^a^	-0.1160	0.2205^a^

^a^ significant at 5%.

From Table 2, latitude was more strongly associated to the variation of the aboveground carbon stock distribution of arboreal vegetation of the Atlantic Forest, Savanna and Semi-arid woodland Biomes in Minas Gerais State. Latitude and altitude presented significant correlation with carbon stock. In contrast, longitude presented a non-significant correlation at the significance level of 5%.

The significant correlation among latitude, altitude and carbon stock is a reflection of the climatic and topographic conditions of Minas Gerais State. Regions of lower latitudes (in South Hemisphere latitude presenting negative values) have lesser average temperature. These locations are more intensely influenced by cold fronts, which weaken as they move north toward highest latitudes by increasing atmospheric pressure, turning them into places where frontal type rain events are more common. Therefore, regions of lower latitudes receive more rainfall in the dry period [26]. It is understood that as latitude decreases, temperature are less, leaving the extreme temperatures of northern region of the State, and presenting greater annual rainfall amounts. Thus, as latitude decreases, higher is the carbon stock. Altitude has a similar behavior, but with less impact.

The accuracy statistics in the parameterized model (as proposed by Eq 6), according to geographical variables significantly correlated to the dependent variable, are presented in Table 3.

**Table 3:** Mean Absolute Error (MAE (%)), determination coefficient (R^2^), residual standard error (Sxy (Mg/ha)), and p-value of the Shapiro-Wilk (SW) test.

MAE (%)	Sxy (Mg/ha)	R²	SW
58.39%	16.21	0.53	0.18

**Fig 4:**
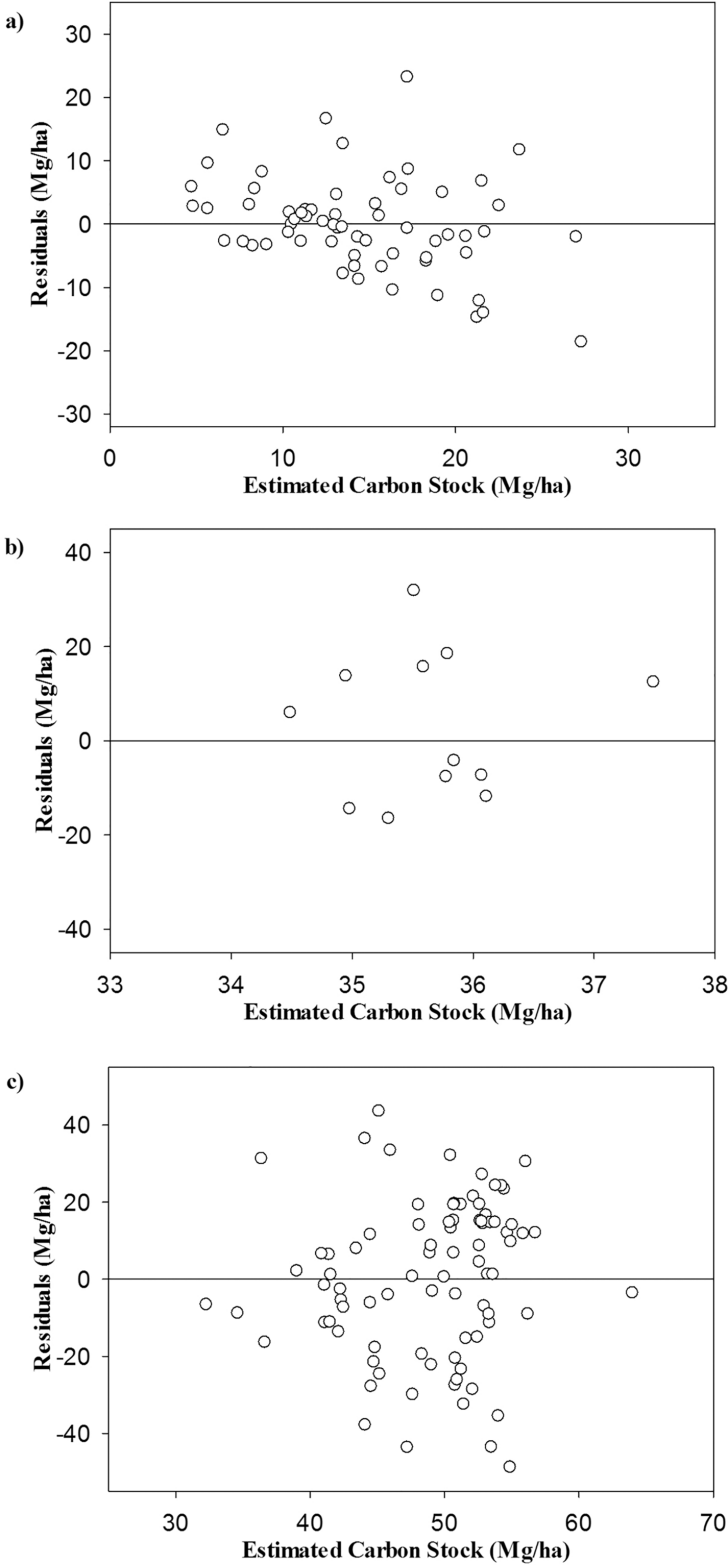
Distribution of the residuals generated by the geographical model for aboveground carbon stock of the arboreal vegetation in the Savanna (a), Semi-arid woodland (b), and Atlantic Forest (c) Biomes of Minas Gerais State.

The parameterized geographical model presented an R² value equal to 53%. This value is acceptable due to the wide variation found in the data. With respect to the Mean Absolute Error (MAE (%)) and Residual Standard Error (Sxy), the model showed a values of 58.39% and 16.21 Mg/ha, respectively. According to p-value of Shapiro-Wilk test, there is no dependence in the residuals, which configures the model free of bias. In addition, in Fig 4, the model presented randomly distributed residuals without a significant trend. For the Savanna (a), Semi-arid woodland (b), and Atlantic Forest (c) Biomes, the residual distribution indicated that the model has no trends toward underestimates or overestimates, indicating no bias of estimates.

Table 4 presents the results of the parameterized geographical model, the average estimated parameters, the Student “t” test for each parameter, the standard error associated with each of them, and the Variance Inflation Factor values. It is necessary to emphasize that in the parameterized model there was no non-significant coefficients and all coefficients showed a high statistical significance.

**Table 4:** Parameterized geographic model with parameters estimated by regression, statistical significance (Student “t” test) of estimated parameters and the Variance Inflation Factors (VIF).

Parameter	Variable	Coefficient	Standard Error	VIF
β0	Intercept	-52.76 ^a^	16.29	1.19
β1	LA	-3.53 ^a^	0.84	1.44
β2	1/A	4160.82 ^a^	2061.91	1.06

AL is the altitude; LA is the latitude; AL and LA in meters and decimal degrees, respectively.

^a^ Significant at 5%.

From Table 4, the minor variance estimate of the parameters may also be highlighted, with high statistical significance of selected input variables. The selected input variables did not present any collinearity, as confirmed by the low variance inflation factors values found [33–34].

The geographic equation for Semi-arid woodland, Atlantic Forest and Savanna Biome can be computed as follows (Eq 8):

$$C=-52.76+25.87\times AF+26.55\times SW-3.53\times LA+4160.82\times \frac{1}{AL}$$ (8)

Where: *C* is carbon stock (Mg/ha); *AF* and *SW* are constants associated with Atlantic Forest and Semi-arid woodland, since Savanna Biome is the intercept baseline; *LA* is latitude in decimal degrees (Albers coordinates); *AL* is altitude in meters.

### Residual Kriging

**Fig 5:**
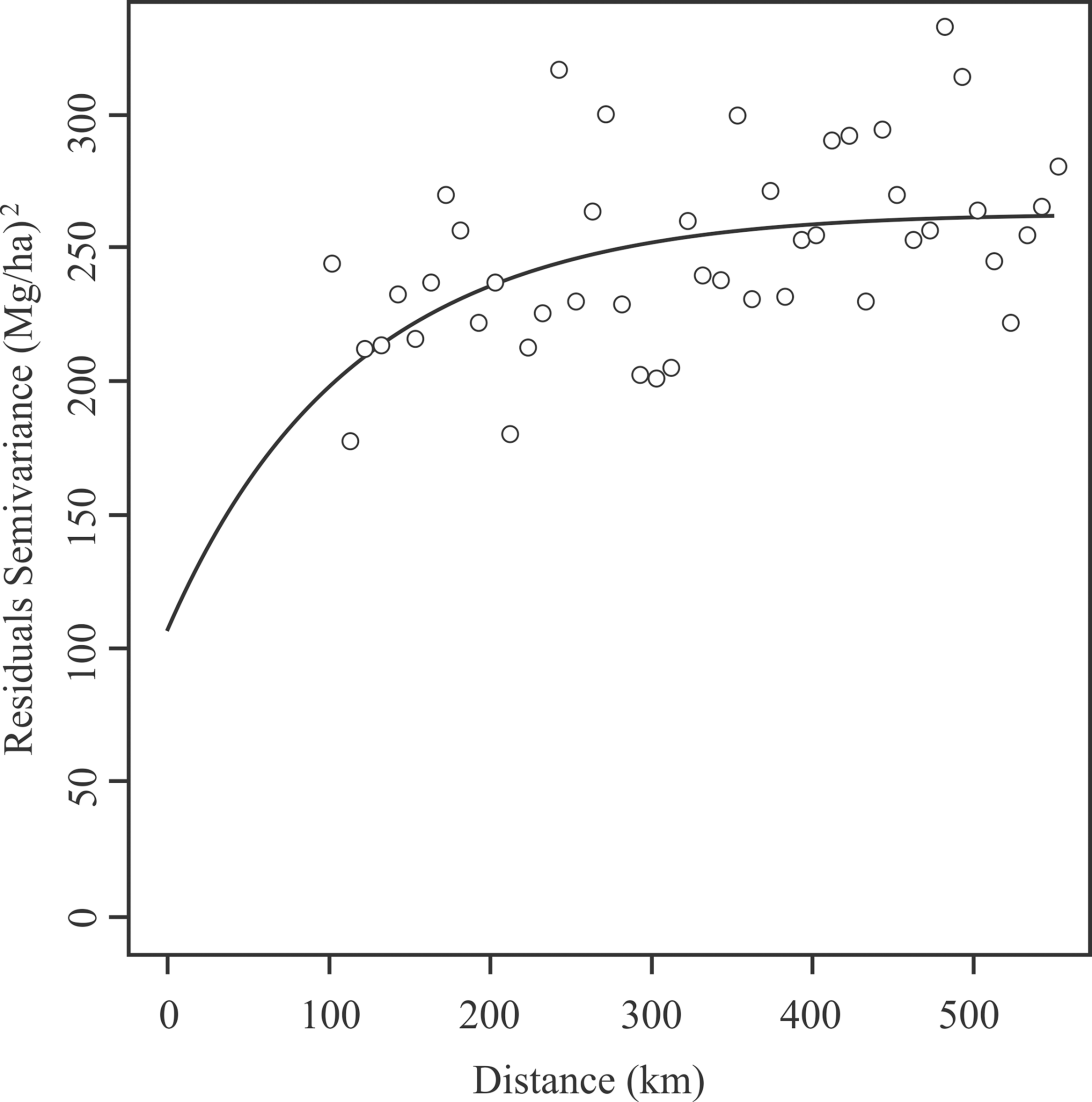
Theoretical and experimental unidirectional semivariogram for the residual aboveground carbon stock of the arboreal vegetation (Mg/ha) of Minas Gerais State Biomes generated by the geographical model.

The experimental and modeled semivariograms for the residues derived from the geographical model (Eq 8) for aboveground carbon stock of the arboreal vegetation of the Biomes of Minas Gerais State are presented in Fig 5. It was possible to evaluate that the exponential model was reasonably parameterized, which allowed for the modeling of the spatial continuity of the variable of interest, showing a range of 339.2 km. The range parameter estimated for the semivariogram is compatible with both the extensive dimension of Minas Gerais State and the density of sampled points. The values of the nugget effect (108.82) and sill (153.78) characterizes a moderate spatial dependency degree [35].

### Mapping of the aboveground carbon stock of the arboreal vegetation of the Biomes of Minas Gerais State and description of the carbon concentration distribution

**Fig 6:**
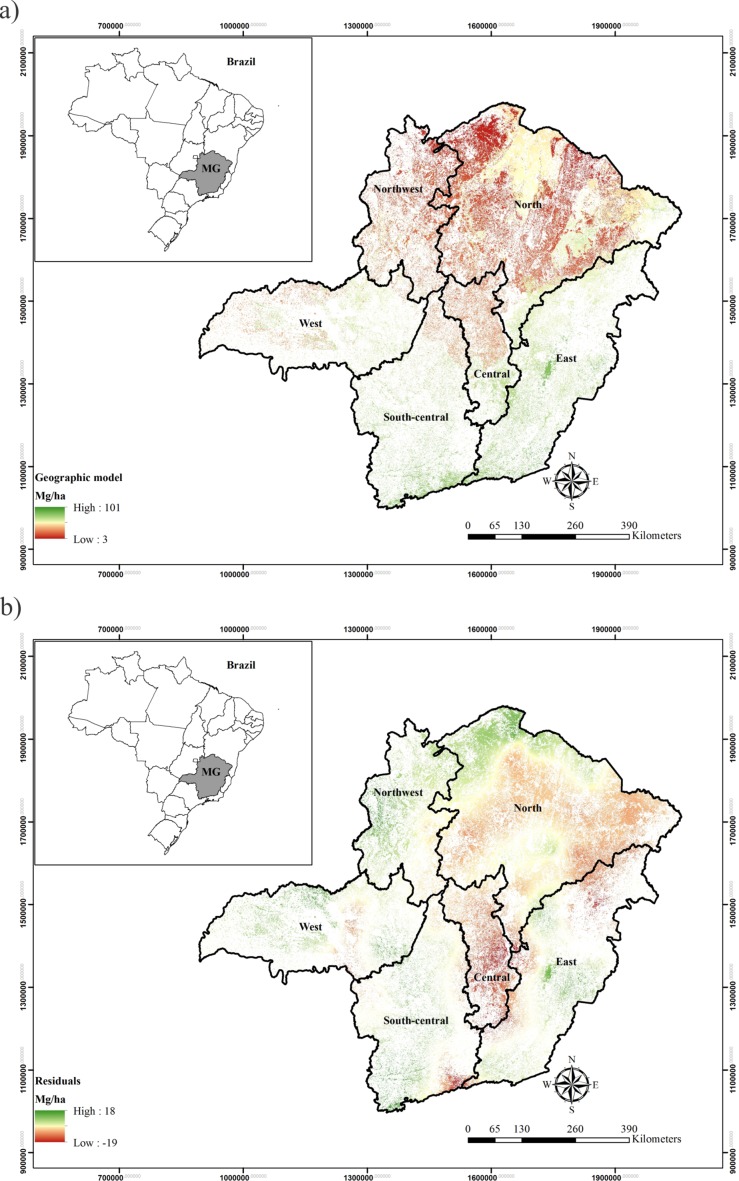
Geographic model (a) and residual Kriging (b) maps for aboveground carbon stocks of the arboreal individuals in the Biomes in Minas Gerais State.

The maps of carbon stock based on the geographical model (a) (global map) and residual Kriging (b) are presented in Fig 6. It is possible to observe some important aspects, besides emphasizing that the sum of these two maps results in the final map for carbon stock by Regression-kriging. First, on the global map, a balance between the underestimates and overestimates can be noticed, which imply that the geographical model is appropriate in its estimates in the study area, as presented and discussed earlier.

Besides this fundamental feature, it is important to highlight in Fig 6 the considerably low estimation values for underestimating (positive values) and overestimating (negative values) of carbon stock values. It demonstrates the good performance not only of the geographical model, but also of ordinary Kriging, because of the expressive spatial continuity modeled for the residuals. When Regression-kriging was used for mapping rainfall erosivity in Brazil and Switzerland [13, 15], respectively, these authors comment on this issue that residuals requires a balanced spatial distribution with no trend for the results of the Regression-kriging to be accurate.

**Fig 7:**
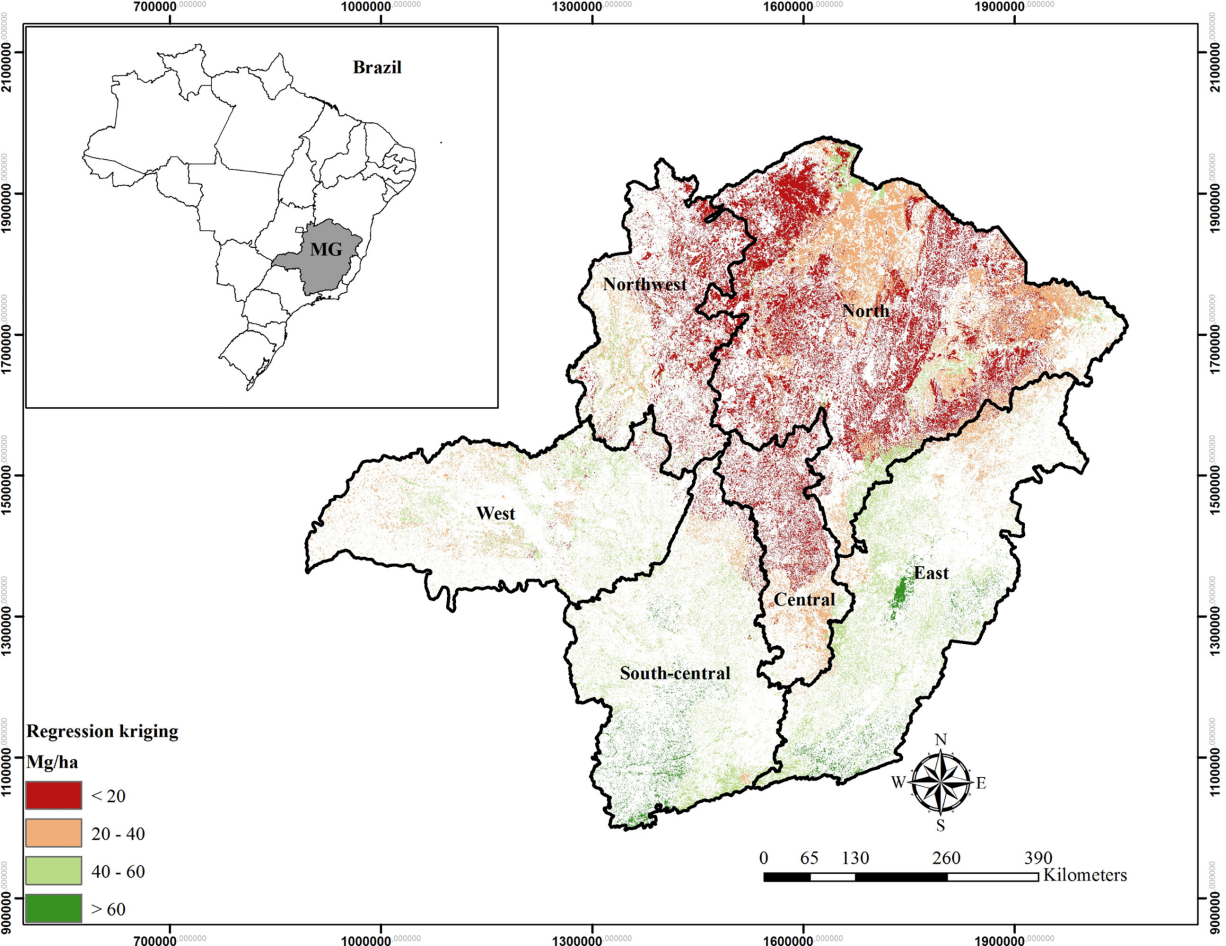
Aboveground carbon stock mapping of the arboreal vegetation of the Biomes in Minas Gerais State obtained by Regression-kriging.

Fig 7 presents the final map of aboveground carbon stock distribution of the arboreal vegetation of the Biomes in Minas Gerais State generated from the hybrid technique of Regression-kriging.

Table 5 presents aboveground carbon distribution in Minas Gerais State, estimated by Regression-kriging procedures. The aboveground carbon estimates of Table 5 were obtained by adding the information presented in Fig 7.

**Table 5:** Average and total aboveground carbon stock of the arboreal vegetation for each Biome, and weighted average for each of the regions in Minas Gerais State.

Region	Biome	Area (ha)	Carbon (Mg/ha)	Carbon (Mg)
Central	Semi-arid woodland	2,556	38.2	97,537
	Savanna	680,767	11.8	8,060,281
	Atlantic Forest	399,869	37.1	14,843,137
	Weighted average and Region Total	1,083,192	21.2	23,000,956
South-central	Savanna	276,226	21.5	5,933,334
	Atlantic Forest	1,166,432	55.0	64,118,767
	Weighted average and Region Total	1,442,658	48.6	70,052,102
East	Savanna	15,941	8.2	130,716
	Atlantic Forest	1,991,150	48.7	96,969,005
	Weighted average and Region Total	2,007,091	48.4	97,099,721
Northwest	Semi-arid woodland	98	35.4	3,469
	Savanna	1,343,184	15.2	20,416,397
	Atlantic Forest	338,354	42.5	14,380,045
	Weighted average and Region Total	1,681,636	20.7	34,799,911
North	Semi-arid woodland	2,127,702	35.1	74,575,955
	Savanna	4,541,992	9.8	44,511,522
	Atlantic Forest	1,222,121	34.6	42,248,723
	Weighted average and Region Total	7,891,815	20.4	161,336,200
West	Savanna	422,872	26.6	11,261,081
	Atlantic Forest	725,275	47.8	34,639,134
	Weighted average and Region Total	1,148,147	40.0	45,900,215
Minas Gerais State	Weighted average and Total for the State	15,254,539	28.3	432,189,104

It is interesting to note that Table 5 presents the total sum of estimated carbon for the entire Minas Gerais State, equal to 432,189,104 Mg. In addition, the total sum of estimated carbon for the Semi-arid woodland, Savanna and Atlantic Forest Biome is equal to 74,676,961 Mg; 90,313,332 Mg; and 267,198,811 Mg, respectively.

Aboveground carbon distribution of the arboreal vegetation in Minas Gerais State in the western region ranges between 20 and 60 Mg/ha, being the band 40–60 Mg/ha the most recurrent. The weighted average of carbon stock for this region was 40.0 Mg/ha (Fig 7 and Table 5). In this region, the phytophysiognomy Densely Wooded Savanna is present, which belongs to the Savanna Biome, in addition to forest remnants from the Atlantic Forest Biome. As confirmed by [18], this region has favorable conditions for plant development, heat, water and nutrient availability, being these factors that directly affect carbon stocking. Located in the central part of Brazil, the western region is affected by the South Atlantic Anticyclone (SAA) weather patterns mainly during the winter, preventing cold front influences during this period [36]. Moreover, there is the influence of the South Atlantic Convergence Zone (SACZ) that according to [36], forms a channel of moisture from the Amazon region, which is responsible for significant events of summer rainfall. The reason that this region does not present a high amount of carbon stock is related to the occurrence of Wooded Savanna vegetation, which is characterized by lower tree density and smaller size of trees (Table 1). Thus, this reduces the carbon average in the region. However, looking specifically at the Savanna Biome, this is the region that possess and which has the greatest carbon stock capacity within the six regions in Minas Gerais State presented in Figs 1 and 7. According to the environmental conditions, the occurrence of the phytophysiognomy Densely Wooded Savanna predominates in the region, which contains larger plants (Table 1).

In the northwest region predominates Savanna vegetation, with transition strips of Densely Wooded Savanna and Wooded Savanna phytophysiognomes. This region has lesser carbon stock with a weighted average of 20.7 Mg/ha (Fig 7 and Table 5). This value may be explained by the occurrence of reduced Atlantic Forest Biome, and greater anthropization. It is also a region with higher temperatures and lesser rainfall [18], which promotes lower carbon stocking.

The vegetation in the northern region of the State is constituted mostly by the phytophysiognomy of Wooded Savanna belonging to the Savanna Biome, followed by Semi-arid woodland and Atlantic Forest Biomes. Wooded Savanna features carbon stock predominantly below 20 Mg/ha, as a result from the combination of temperatures exceeding 25°C and low annual rainfall (850 mm). It features a sub-humid or even semi-arid climate, resulting in environmental stress that is reflected in the plant development. Another relevant point is the high social vulnerability of the local population, which increases illegal vegetation extraction in the area, and thus, affecting the development of remnants (forest fragments). As for the Semi-arid woodland Biome, despite being in the same north region, it contains predominantly arboreal-sized vegetation (Table 1) with higher carbon stocks than the Wooded Savanna [37]. It is also noted that this region has showed the lowest carbon stock average for the Atlantic Forest Biome, being a reflection of the less favorable edaphoclimatic conditions of this region in relation to the others.

In the central region of Minas Gerais State there is predominance of the Savanna Biome, with a more significant presence of Wooded Savanna and Shrub Savanna phytophysiognomes. The last phytophysiognomy occurs further north, occupying a fraction larger than the area of Wooded Savanna. Considering the phytophysiognomes that are part of this study, this one has a lower plant density per hectare and smaller size (Table 1), the weighted average of the Savanna Biome is low, equal to 11.8 Mg/ha (Fig 7 and Table 5). From the central to the southern strip of this region, carbon stocks range from 20 to 40 Mg/ha, a fact explained by the presence of the Atlantic Forest Biome and in a tiny area, the Semi-arid woodland Biome (Fig 7 and Table 5). This region features concentrated rainfall for three months of the year (1250 mm) and strong anthropic pressure, which reflects negatively on remnants development.

In the eastern and south-central regions of Minas Gerais State, carbon stock ranged from medium-high to high, with the predominant of the range 40–60 Mg/ha, and several bands greater than 60 Mg/ha. In these regions, the weighted averages are 48.4 and 48.6 Mg/ha, respectively (Fig 7 and Table 5). Because they are areas with a predominance of forest remnants of the Atlantic Forest Biome, they have a greater carbon accumulation. Its remnants have high plant density (Table 1) and large numbers of vigorous plants. These regions also present better rainfall distribution and total annual rainfall greater than 1500 mm. In addition, the south-central region receives a considerable influence from the SACZ in summer and cold fronts throughout the year, and the eastern region, close to the coastal ranges of Brazil, are areas with good rainfall amounts, favoring the development of forest remnants.

Seeking to understand the carbon stocking associated with the agro-industrial context of Minas Gerais State, it was observed that the search for enhancement and maintenance of forest remnants in the central, south-central, eastern, western, and more recently northwestern regions are a major challenge. These regions have been subjected to high historical anthropic action [38], combined with high dependence of the State for agricultural production and animal husbandry. These regions also lack realistic public policies focused on food production and conservation of native resources, so the regions already present a significant area of degraded soils.

In particular, in the eastern and south-central regions, remnants of native flora have good rates of carbon stocking because they are predominantly of the Atlantic Forest Biome. The existing remnants are concentrated primarily in mountain range areas, such as Serra da Mantiqueira, in hilltops [39], and in small and medium-sized forest fragments with areas systematically less than 100 ha. The conservation of the existing vegetation, coupled with effective revegetation programs of Legal Reserve areas, which corresponds to 20% of the area of each rural property [40] will bring major environmental benefits for the State, for Brazil, and for the planet. Also, the recovery of permanent preservation areas (foreseen in the forest and environmental management legislation), directly linked to the protection of water sources and complementary public policies, will result in large gains in water yield, fauna protection, erosion control, more sensible handling of hydrographic microbasins, CO_2_ capture and carbon stocking, and avoiding competition with food production, essential for the well-being of the population.

The western region is largely favorable for agro-industrial development in Minas Gerais State [41]. That region has been exploited for the production of grains and beef cattle [42] because it has a high production capacity, by combining good soils in agronomic terms and a favorable climate. Therefore, in addition to being a region that presents good carbon stocking by the predominance of Atlantic Forest and Densely Wooded Savanna remnants, it still lacks native vegetation to promote adequate protection of fauna and particularly for streamflow generation for rivers.

The central, northern, and northwestern are regions characterized by unproductive sites [43], thus, these are regions must seek technology-based development, switching labor from agricultural to technological. In doing so, it could focus on the conservation of native vegetation and more sustainable practices, such as ecological tourism, given its scenic beauties. [8, 44] studies have shown that there is strong pressure for an alternative use of the soil in these regions; since the largest vegetation areas in Minas Gerais State are concentrated in Savannas, which make up to the greatest proportion of vegetation and currently suffer the greatest deforestation rates.

## Conclusions

The geographical model generated for the existing native vegetation in Minas Gerais State, Brazil, showed that Biome, latitude and altitude are variables capable of explaining changes in aboveground carbon stock in different regions of Minas Gerais State.

The map generated by the hybrid interpolation method of Regression-kriging captured details whose accuracy is supported by a homogeneous residual distribution, which resulted in a consistent distribution of aboveground carbon stock for the Atlantic Forest, Semi-arid woodland, and Savanna Biomes.

In the Atlantic Forest Biome, the greatest concentration of aboveground carbon stock was observed in the south fraction of the eastern region and in the south-central region, a location with the highest weighted averages of carbon stock per hectare.

The lowest weighted average of carbon stock per hectare occurred in the Savanna Biome, particularly in the central, northern, and northwestern regions of Minas Gerais State.

The largest contingent of native vegetation is found in the northern region of Minas Gerais State, a region containing the three Biomes under study. In this region, we found the largest carbon stock among all six regions under study, a fact that requires differentiated conservation policy for this region.
